# Block Partitioning Information-Based CNN Post-Filtering for EVC Baseline Profile

**DOI:** 10.3390/s24041336

**Published:** 2024-02-19

**Authors:** Kiho Choi

**Affiliations:** 1 Department of Electronics Engineering, Kyung Hee University, Yongin-si 17104, Gyeonggi-do, Republic of Korea; aikiho@khu.ac.kr; 2 Department of Electronics and Information Convergence Engineering, Kyung Hee University, Yongin-si 17104, Gyeonggi-do, Republic of Korea

**Keywords:** EVC, MPEG-5, video coding standard, post-filtering, CNN

## Abstract

The need for efficient video coding technology is more important than ever in the current scenario where video applications are increasing worldwide, and Internet of Things (IoT) devices are becoming widespread. In this context, it is necessary to carefully review the recently completed MPEG-5 Essential Video Coding (EVC) standard because the EVC Baseline profile is customized to meet the specific requirements needed to process IoT video data in terms of low complexity. Nevertheless, the EVC Baseline profile has a notable disadvantage. Since it is a codec composed only of simple tools developed over 20 years, it tends to represent numerous coding artifacts. In particular, the presence of blocking artifacts at the block boundary is regarded as a critical issue that must be addressed. To address this, this paper proposes a post-filter using a block partitioning information-based Convolutional Neural Network (CNN). The proposed method in the experimental results objectively shows an approximately 0.57 dB for All-Intra (AI) and 0.37 dB for Low-Delay (LD) improvements in each configuration by the proposed method when compared to the pre-post-filter video, and the enhanced PSNR results in an overall bitrate reduction of 11.62% for AI and 10.91% for LD in the Luma and Chroma components, respectively. Due to the huge improvement in the PSNR, the proposed method significantly improved the visual quality subjectively, particularly in blocking artifacts at the coding block boundary.

## 1. Introduction

The current growth in global video applications, driven by consumer desire for high-quality experiences, has expanded the relevance of devices dramatically [1]. This spike has resulted in a significant increase in frame rates per second to support natural motion, leading to an increase in video content capacity. According to Cisco statistics [2], video-related traffic accounts for around 80% of overall Internet traffic, highlighting the widespread relationship between data transmission and video content. This trend is not confined to 2D movies; it includes 3D videos, volumetric stereoscopic images, 360-degree videos, and VR/AR material, all of which require more data capacity [3].

At the same time, the increase in Internet of Things (IoT) systems has increased the need for effective video coding technology [4]. The significant growth of video data in IoT systems pursues dedicated coding and processing methods. As these systems focus on local data processing for intelligent sensor nodes, the importance of minimizing data volume while ensuring high-quality decoded images becomes important [5]. Thus, video coding technology has importance in nodes that extend beyond traditionally used areas, including small edge units of computing.

Traditionally, video compression technology has been developed through standards created by organizations such as the ISO/IEC Moving Picture Experiences Group (MPEG) and ITU-T Video Coding Experiences Group (VCEG). Standards such as MPEG-2/H.262 [6], Advanced Video Coding (AVC)/H.264 [7], and High Efficiency Video Coding (HEVC)/H.265 [8] have contributed significantly to the efficient compression and transmission of video data. Recently, new video coding standards such as Versatile Video Coding (VVC)/H.266 [9] and MPEG-5 Essential Video Coding (EVC) [10] have been introduced. While VVC/H.266 was developed jointly by the MPEG and VCEG, EVC is a product exclusively for the MPEG.

In this context of the growth of video data in IoT systems, it is necessary to carefully review the completed EVC standard. In particular, the EVC Baseline profile is customized to meet the specific needs of handling IoT video data. Since this profile aims to build a royalty-free codec using conventional coding techniques that are more than 20 years old, focusing only on performing key functions, avoiding the integration of complex tools, results in high-performance compression even at a low complexity [11]. Therefore, it is believed that the EVC Baseline profile proves to be a proper video codec for sensor node networks that require high-performance compression while operating at low power and complexity, and it is expected to play a pivotal role in addressing the growing need for high performance within the IoT ecosystem.

However, the EVC Baseline has a notable drawback. Being a codec comprised solely of simple tools developed over 20 years, it tends to exhibit numerous coding artifacts. Specifically, the presence of blocking artifacts at the block boundaries is considered a critical issue that needs resolution. To address this, a post-filter leveraging block partitioning information-based Convolutional Neural Network (CNN) is introduced in this paper. The proposed filter aims to rectify the challenges associated with the EVC Baseline profile, characterized by a high occurrence of coding artifacts. The proposed post-filter seeks to provide a high-efficiency compression performance and enhanced image quality, making it suitable for node sensor networks with low-complexity requirements. The main contributions of this study can be summarized as follows:

(1) A CNN-based post-filter for the EVC Baseline profile was developed, offering a promising video coding solution for IoT devices.
(2) An analysis of the major artifacts in the EVC Baseline profile was conducted, and a method indicating the area where these artifacts appear was exploited.
(3) The incorporation of a guide map based on blocking partitioning information was implemented to identify attention areas and enhance visual quality in the target image and video.
(4) Consideration was given to IoT applications with low complexity, allowing IoT devices to selectively add the post-filter based on the available extra computing power.
(5) A scenario-based CNN-based post-processing network was developed for real IoT applications, whether in image-based or real-time broadcasting/streaming services.

The remainder of this paper is organized as follows. Section 2 provides an overview of the EVC Baseline profile, related works of CNN-based filtering technologies, and standard activity. The proposed method is presented in Section 3. Section 4 provides an overall performance evaluation and analysis. Finally, Section 5 concludes this paper.

## 2. Related Work

To examine the relevant work of the proposed method, this section initially offers background information on EVC Baseline profiles. Following that, it explores CNN-based filtering for video coding, encompassing both in-loop filtering and out-loop filtering. Finally, it will outline the recent developments in standards for neural network-based video coding at the Joint Video Exploration Team (JVET), a collaboration between the MPEG and ITU-T.

### 2.1. Overview of EVC Baseline Profile

The block structure of the EVC Baseline profile is based on a partitioning method that supports quadtree division based on 64 × 64 blocks. The maximum block size of the coding unit is 64 × 64 and the minimum size is 4 × 4. For intra prediction, the process is performed based on the coding unit block, incorporating five supported prediction modes. The intra prediction supported by the Baseline profile would be impossible to accurately predict direction, but the major intra directionality can be predicted, thereby reducing the redundancy of directional information within a frame.

The residual value generated in the prediction process is converted into a frequency value through Discrete Cosine Transform (DCT), and the converted coefficient value is converted into a quantized coefficient value through a quantization process. The size of the transformation aligns with the size of the prediction block, and a process with a quantization parameter (QP) in the range of 0 to 51 is used for quantization. After the quantization process, the quantized coefficient values are scanned through a zigzag scan order and are then binarized through a basic run-level coding method, and the binarized values are streamed to the entropy coding engine as described in JPEG Annex D [12]. In the case of the filtering tool in the Baseline profile, an initial version of the deblocking filter in AVC/H.264 was applied to improve the objective and subjective image quality. The method is the same as that applied to H.263 Annex J [13].

For the EVC Baseline profile, it has been reported that it achieves approximately 30% bit savings in the objective evaluation and about 40% in the subjective evaluation compared to AVC/H.264, which is widely utilized on the Internet, while maintaining the same quality [14]. Moreover, in terms of complexity, it exhibits one-fourth of the algorithmic complexity when compared to AVC/H.264, making it a promising candidate as an optimal compression codec for next-generation sensor nodes [15].

### 2.2. CNN-Based Filtering Technologies for Video Coding

To improve coding artifacts during the encoding and decoding process, the latest video coding standard comes equipped with an in-loop filter designed. In the case of the VVC/H.266 standard, it incorporates three traditional in-loop filters: Deblocking Filter (DBF), Sample Adaptive Offset (SAO), and Adaptive Loop Filter (ALF). These filters are sequentially applied to the reconstructed frames. The DBF focuses on suppressing blocking artifacts at the block boundaries, while the SAO filter and the ALF aim to eliminate artifacts resulting from quantization. Despite the effectiveness of these filters, there is still considerable room for improvement in terms of visual quality.

Recently, developments have seen an active pursuit of research aimed at minimizing video coding artifacts using neural networks. That research focuses primarily on two aspects: (1) the design of a filter using a neural network for an in-loop filtering method applicable within the codec, similar to the DBF, SAO, and ALF, and (2) the investigation of a post-filter method that can be selectively applied outside the codec as needed.

Park et al. [16] introduced a CNN-based In-Loop Filter (IFCNN) capable of replacing the SAO in HEVC/H.265, and the proposed IFCNN showed a promising coding performance on Bjontegaard Delta bitrate (BD-BR) [17], with reductions of 2.6% and 2.8% for the Random-Access (RA) and Low-Delay (LD) configurations, respectively. Dai et al. proposed a Variable Filter Size Residual Learning Convolutional Neural Network (VRCNN) [18], designed to replace conventional filters in HEVC/H.265, such as the DBF and SAO, in HEVC/H.265. The proposed method in [18] utilized the variable block size of transform in HEVC/H.265; thus, residual learning led to faster convergence. According to [18], the VRCNN reduced the BD-BR by an average of 4.6% in the All-Intra (AI) configuration. Similar to the motivation of earlier methods, Kang et al. introduced a multi-scale CNN (MMS-net) [19] that could replace the DBF and SAO in HEVC/H.265 by utilizing skip connections with different scales from subnetworks to enhance the restoration process. The proposed MMS-net’s performance on the BD-BR showed a reduction of 8.5% for the AI configuration. Wang et al. [20] proposed an attention-based dual-scale CNN (ADCNN), which utilized the encoding information, such as the QP and partitioning information, and the proposed ADCNN’s performance on the BD-BR showed reductions of 6.5% and 2.8% for the AI and RA configurations, respectively. The residual highway CNN (RHCNN) in [21] utilized residual units with a progressive training scheme for the QP bands, and the proposed RHCNN’s performance on the BD-BR showed reductions of 5.7%, 4.4%, and 5.7% for the AI, RA, and LD configurations, respectively. Similar to the approach of [21], Wang et al. [22] applied a neural network-based in-loop filter (CNNLF) in the conventional video coding framework in VVC/H.266 by conducting the modules of feature extraction and image quality enhancement. Compared with VTM-15.0, the proposed CNNLF improved the PSNR by 0.4 dB and 0.8 dB at 0.1 Mbps, respectively, and by 0.2 dB and 0.5 dB at 1 Mbps, respectively. Huang et al. [23] also added the CNN-based network to the conventional video coding framework specifically between the DBF and SAO in VVC/H.266. The proposed method based on a variable CNN utilized an attention module into a residual block to extract informative features, and the proposed method in [23] showed reductions of 3.6%, 3.6%, and 4.2% in performance on the BD-BR for the AI, RA, and LD configurations, respectively.

The purpose for post-filtering approaches is similar to that of in-loop filtering; however, it is used outside of the codec architecture. Thus, CNN-based post-filtering algorithms can be selectively applied to decoded images to improve visual quality. Dong et al. [24] introduced a CNN-based artifact removal method (AR-CNN) designed for JPEG compressed images, which was an extension of the super-resolution CNN (SRCNN) from previous studies. The results presented in [24] demonstrated a 1 dB improvement achieved by the proposed AR-CNN when compared to JPEG images. Li et al. [25] presented a method employing a twenty-layer CNN architecture with residual learning. An interesting aspect of the method proposed in [25] involved transmitting side information related to video content complexity and quality indicators from the encoder to the decoder at each frame. The performance of the method, as reported in [25], demonstrated a 1.6% BD-BR reduction compared with HEVC/H.265 on the six sequences given in the 2017 ICIP Grand Challenge. Zhang et al. [26] introduced a post-processing architecture based on a CNN for VVC/H.266 compressed video sequences. This architecture utilized 16 identical residual blocks and incorporated three types of skip connections, and it was reported that the proposed method in [26] showed a reduction of 3.9% in performance on the BD-BR for the RA configuration compared to VVC/H.266. The authors extended the [26] method, incorporating a generative adversarial network (GAN)-based training strategy to improve the visual quality of VVC/H.266-decoded images. The proposed method in [27] showed a notable enhancement in perceptual visual quality, achieving a reduction of 3.9% in performance on the BD-BR for the RA configuration compared to VVC/H.266. Bonnineau et al. [28] introduced a multitask learning-based approach that employed a QP map to generalize the model with various QPs by sharing parameters within a single network and task-specific modules. The method presented in [28] exhibited a significant improvement in perceptual visual quality, achieving a reduction of 2.8% in performance on the BD-BR for the RA configuration compared to VVC/H.266. Wang et al. [29] aimed to enhance the visual quality of decoded images by incorporating partitioning information with QP information, introducing a three-branch network. The method described in [29] demonstrated a notable improvement in perceptual visual quality, achieving a reduction of 6.5% in performance on the BD-BR for the AI configuration compared to VVC/H.266. Meng et al. [30] presented a network for enhancing visual quality, combining temporal motion and spatial information through a fusion subnet and an enhancement subnet. The approach outlined in [30] showed a significant improvement in perceptual visual quality, achieving a 0.29 dB enhancement compared to VVC/H.266-decoded images.

### 2.3. Neural Network-Based Video Coding

Meanwhile, various applications have recently explored the advancement in neural network (NN) technology. For instance, machine learning is leveraged in natural language processing and computer vision to overcome performance barriers. This trend is also making an impact on the development of video coding. The JVET is actively monitoring the adoption of NN technology and has initiated research into Neural Network-based Video Coding (NNVC) [31]. During the 130th MPEG meeting and 19th JVET meeting, two independent Ad Hoc Groups (AHGs) related to NNVC were formed, both focusing on the development of (1) an end-to-end (E2E) video coding framework and (2) the integration of NN in a hybrid video coding framework. Subsequently, these two AHGs were consolidated under the JVET, with the merged group tasked with assessing the feasibility of NNVC for potential coding gains compared to traditional video coding standards based on signal processing technology. Currently, the development of in-loop filtering mainly using neural networks is being actively discussed in the JVET. It should be noted that in JVET activities, the main architecture of the network is based on a res-block CNN structure. Considering the fact that video coding generally uses a residual-based encoding/decoding approach that relies on accurate predictions about intra/interframes, the focus for improvement is mainly on preserving the details expressed through content distribution without changing the DC value. Thus, this approach, using the res-block basis CNN architecture, aligns well with the overall architecture of video coding, proving effective for in-loop filtering.

## 3. CNN-Based Post-Filtering with Block Partitioning Information

In the previous section, we reviewed the filtering technologies employed in conventional video coding standards and the recently emerged neural network-based filtering methods. While the future outlook for neural network-based filtering technologies appears promising, it is acknowledged that they still present challenges in terms of complexity. Given this context, one might argue that a post-filter, capable of adaptively enhancing image quality as needed, is more practical than an in-loop filter, which must be consistently applied to sensor nodes requiring fast processing with low complexity. Therefore, this paper proposes a CNN-based post-filter for EVC, aiming to enhance the image quality and compression rates while maintaining the constraints of low power and low complexity.

### 3.1. Analysis of Coding Artifacts

**Figure 1:**
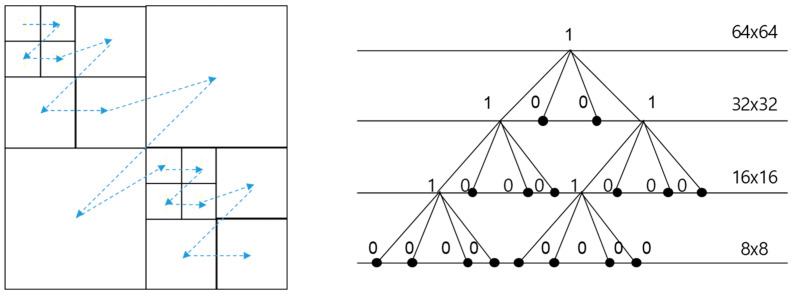
Quadtree-based coding structure in EVC Baseline profile.

The EVC Baseline profile employs a quadtree-based coding structure, allowing the utilization of blocks up to 64 × 64, as illustrated in Figure 1. This method involves determining the optimal block size through processing from 64 × 64 to 4 × 4 in the encoder and transmitting this information to the decoder based on the quadtree. For example, during the decoding process, if the split flag is 0, the coding block for the process is 64 × 64. If the split flag is 1, four additional split flags are transmitted, indicating whether the coding block should be divided into units of 32 × 32. This process continues until the information is transmitted down to 4 × 4, a leaf node. The size of the coding block is determined according to the characteristics of the content, and specifically, the coding block is determined as a large block in homogeneous areas and a small block in delicate areas. Nevertheless, while the EVC block decision process ensures optimal rate–distortion (RD) performance, the absence of high-performance in-loop filtering in the EVC Baseline profile leads to the generation of significant artifacts around the block.

**Figure 2:**
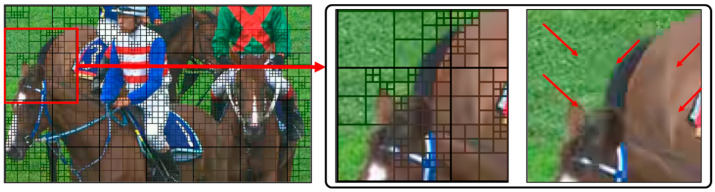
Example of coding artifacts detected in the area of the block boundary encoded with the EVC Baseline profile at the RaceHorses sequence with QP = 37.

Errors in the video coding process include ringing artifacts, blocking artifacts, and bending artifacts. Among these, the most noticeable artifact for video consumers is the blocking artifact, primarily occurring at the block boundaries in block-based video coding. Specifically, in the EVC Baseline profile, the discontinuity at the block boundary is pronounced, leading to a significant degradation in the image quality of the decoded image. Figure 2 shows an example of the result of encoding by the EVC Baseline profile on QP = 37 to the RaceHorses sequence, clearly showing the prominent presence of blocking artifacts at the block boundary. The problem is that the Baseline profile contains an excessive number of such blocking artifacts. To address this concern, our research aims to improve the visual quality of the decoded images produced by the EVC Baseline profile. This improvement is accomplished by employing a block partitioning strategy within the context of CNN-based post-filtering.

### 3.2. Architecture and Network

**Figure 3:**
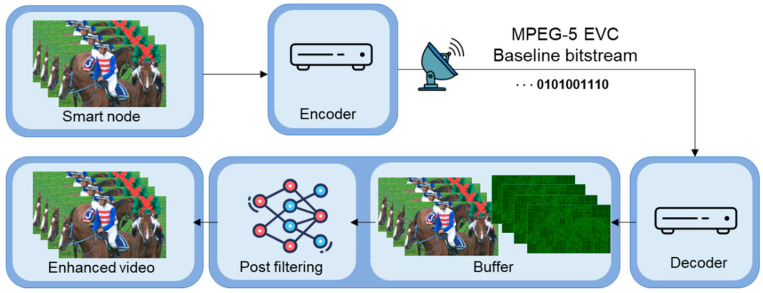
The overall pipeline for applying the proposed post-filtering in the use case.

Figure 3 depicts the overall pipeline for applying the proposed filtering in this paper. As depicted in the figure, in the case of this proposed post-filter, a filtering process is performed in the out-loop with the decoded image of the EVC Baseline profile. The CNN-based post-filter takes the decoded image and the block partitioning information extracted during the decoding process as the input and then improves the image quality by passing it through the trained CNN model.

**Figure 4:**
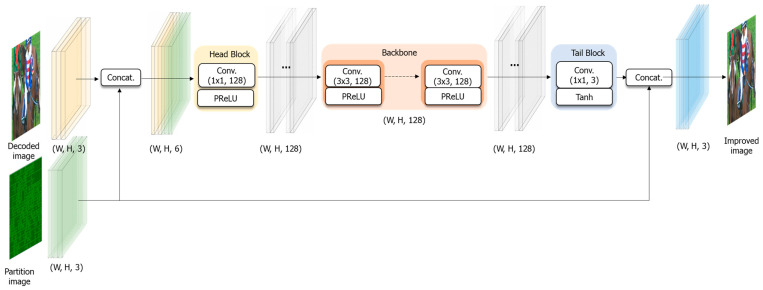
Proposed CNN-based post-filtering with block partitioning information.

The architecture in our proposed method is an extension of [32]. In the previous work, we utilized QP map information in the context of CNN-based post-filtering targeting the VVC/H.266 standard. In this paper, we extended this concept by integrating the block partitioning information into CNN-based post-filtering, specifically targeted for the EVC Baseline profile, which is suitable for video data transmission in sensor nodes. Figure 4 outlines the comprehensive network design employed in our proposed method.

In the initial processing block, the decoded image from the EVC Baseline profile is combined with a block partitioning map and framed. Both the decoded image from the EVC Baseline profile and the block partitioning map operate based on YUV channels. After concatenation, the number of input channels doubled. This package uses the decoded image from the EVC Baseline profile as the target for improvement, and the block partitioning map guides the areas with artifacts in the target decoded image.

The packaged video is then fed into the head block, comprising a 1 × 1 convolution filter with 128 output channels and a Parametric Rectified Linear Unit (PReLU). The primary function of the head block is to decrease the input dimensions for the subsequent backbone blocks. In this head block, we configured 128 channels to generate multiple feature representations, and the resulting 128 channels undergo the activation function (i.e., PReLU) to apply non-linearity to the output of the head block.

The features extracted from the head block are then directed to the backbone blocks, which encompass multiple blocks focused on extracting features. In the fundamental feature extraction block, a 3 × 3 convolution filter with 128 output channels and a PReLU is employed. The 3 × 3 convolution filter plays a crucial role in extracting features from the input and generating essential features for the subsequent layers. The 128 output channels from the 3 × 3 convolution filter undergo the PReLU activation function, and this process is repeated through the layers up to the final feature extraction block to ensure convergence in the deeper layers of the network.

In the proposed method described in this paper, we utilized 16 feature blocks based on empirical studies, but this number can be adjusted depending on the characteristics of the input decoded image. To maintain network simplicity, we designed the backbone block with a shape similar to the head block. While the head block primarily reduces the input dimensions, the backbone block focuses on capturing residual features for training.

The tail block, responsible for processing the output channels from the backbone blocks, integrates a 1 × 1 convolution filter with three output channels and employs the Tanh activation function, replacing the PReLU. To achieve precise quality improvement, we chose to update the residuals of the decoded image. As a result, the input decoded image from the head block is connected to the output of the tail block through a skip connection. The residual updates ensure that the primary values of the decoded image remain unaltered, while enabling adjustments to the corrupted areas introduced during the encoding and decoding processes, which represent the core objective of the proposed method.

### 3.3. Training

To create the training dataset, we utilized the BVI-DVC [33] dataset, comprised of 800 videos of varying resolutions ranging from 270 p to 2160 p, providing a diverse set of training data. Given that the BVI-DVC dataset is based on mp4 files, we converted these files to the YUV420 format with 10-bit files for the training dataset using FFmpeg [34]. To streamline the dataset creation, we extracted 10 frames from each video, resulting in a training dataset of 8000 frames. Ensuring uniform sizes for each Y, U, and V channel, we upsampled the U and V channels to match the size of the Y channel. Following the conversion, the original YUV format videos were processed through XEVE [35] and XEVD [36] to produce decoded images in the YUV format. Subsequently, instead of utilizing the entire image size for the training dataset, we cropped each image from the original and decoded YUV images to a size of 256 × 256. We then randomly selected the cropped images produced through horizontal and vertical flipping processes.

For the models in the proposed method, we generated five models corresponding to the QP values. Standard groups, such as the MPEG and JVET, use a common test condition (CTC) for experiments to evaluate suggested contributions, usually utilizing four or five QP values. A QP value in a codec plays an important role in this process. An increase in the QP results in higher distortion due to a coarser quantization step applied to transform coefficients with a larger QP. This leads to the loss of high-frequency information and a broader distribution range for the compensation value between the reconstructed and original pixels. Conversely, a low QP value yields better visual quality but requires a relatively high bitrate. Therefore, the QP number serves as a fundamental control parameter determining the visual quality and bitrate of the video. 

In the proposed method, the utilization of models dependent on the QP is a critical aspect contributing to the generation of high-quality outputs. During the training process, we generated five bitstreams and reconstructed YUV files, depending on the QP value, in accordance with the experiments carried out by the JVET CTC [31]. Subsequently, the proposed model was trained using these five bitstreams and reconstructed YUV files independently. Additionally, we generated models based on different configurations as well. Acknowledging that error characteristics vary with the QP and configuration, we developed a strategy to customize each model to specific error characteristics. The separate models for each scenario in the proposed approach are to ensure that the model is tailored to the specific requirements of each scenario. For instance, the AI model is trained for image-centric applications, while the LD model is designed for real-time broadcasting and streaming purposes. More detailed information on the training process is available in Table 1.

## 4. Experimental Results and Discussion

To evaluate the effectiveness of the proposed method, the JVET CTC [37] sequences were chosen for evaluation but were not included in the training dataset. These 19 sequences were classified into classes A1, A2, B, C, and D based on their resolution with characteristics. The test QP values for all configurations were 22, 27, 32, 37, and 42, corresponding to the JVET CTC. Given the potential applications for the EVC Baseline profile with CNN-based post-filtering, which could be used for a low-complexity, low-power sense node for video data transmission, we evaluated the proposed method using AI and LD configurations. Table 2 contains detailed test sequences and conditions.

### 4.1. Objective Testing Result

For the objective evaluation, the increase in the PSNR was measured at the same bitrate of each sequence over all the QP values (i.e., BD-PSNR) [37], and the BD-BR was also measured to check on the bitrate reduction at the same visual quality that is usually used in standard experiments. Table 3 and Table 4 present the experimental results of the proposed method for the AI and LD configurations, comparing the decoded image of the EVC Baseline profile to the enhanced image filtered by the CNN-based post-filter. The numbers in the table represent the average bitrate and PSNR of the five QPs of each sequence in the reference and proposed method.

Those metrics, the BD-PSNR and BD-BR, compare the improvement in the PSNR and coding efficiency of different video codecs or encoding settings while taking into consideration both the bitrate and video quality. The fundamental concept involved fitting a cubic polynomial curve through five data points and subsequently deriving an expression for the integral of the curve. The BD-PSNR allows for an objective assessment of PSNR improvement by calculating the difference in the PSNR to achieve a comparable bitrate between two codecs. In the BD-PSNR, a higher number indicates an improvement in the PSNR over the anchor. Similarly, by measuring the difference in the bitrate needed to attain an equivalent quality level between two distinct codecs, the BD-BR metric facilitates an objective assessment of the compression efficiency. The lower BD-BR value signifies a higher coding efficiency than the anchor at the same visual quality.

Table 3 shows the results of the proposed method compared to the reference in the AI configuration. As shown in Table 3, the proposed method increases the PSNR at the same bitrate by approximately 0.57 dB, 0.75 dB, and 0.95 dB for the Luma and Chroma components in the AI configuration when compared to the post-filter pre-processed video. The increased PSNR results in overall BD-BR reductions of 11.62%, 24.5%, and 28.79% for the Luma and Chroma components, respectively, in the AI.

Table 4 shows the results of the proposed method compared to the reference in the LD configuration. Similar results can be observed in the LD configuration. As shown in the table, the proposed method increases the PSNR at the same bitrate by approximately 0.37 dB, 0.82 dB, and 0.95 dB for the Luma and Chroma components in the LD configuration when compared to the post-filter pre-process video. The improved PSNR results in overall BD-BR reductions of 10.91%, 31.22%, and 32.30% for the Luma and Chroma components, respectively, in the LD.

The objective test results show that the proposed method significantly improved the visual quality of the decoded images of the EVC Baseline profile, regardless of the QP or configuration. Notably, the proposed method outperforms the AI configuration in terms of a low resolution and high QP values. The main reason for the significant improvements can be attributed to areas where blocking artifacts noticeably appear. Blocks in the AI configuration typically determine the number of coding blocks with a small coding block size; increasing the number of coding blocks results in more blocking artifacts in the block boundary area. This phenomenon can also be applied to the output of low-resolution sequences. Because of the high amount of quantized values for en/decoding, the artifact can be widely visible in coding blocks regardless of the coding block size or number. As a result, the improvements at high QP sequences would be due to an improvement in visual quality across the entire decoded image.

### 4.2. Subjective Testing Result

**Figure 5:**
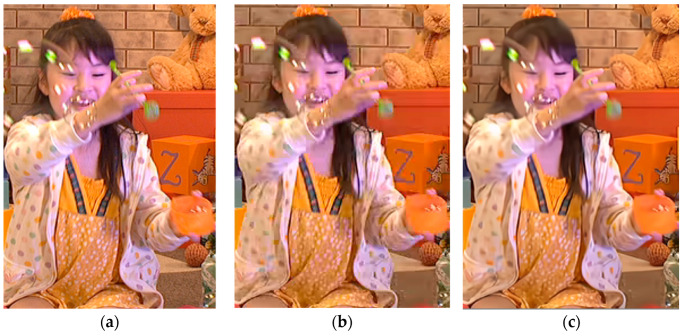
Visual quality comparison with AI configuration at PartyScene with #0 frame: (**a**) original image, (**b**) decoded image, (**c**) proposed method.

**Figure 6:**
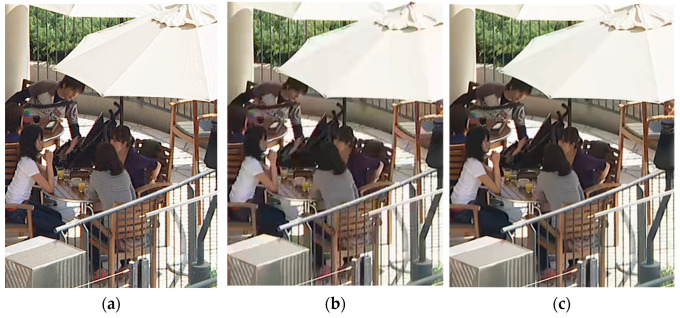
Visual quality comparison with LD configuration at BQTerrace with #11 frame: (**a**) original image, (**b**) decoded image, (**c**) proposed method.

To assess the improvement in visual quality achieved by our proposed method, individual visual quality evaluations were conducted. Figure 5 and Figure 6 present a comparative analysis of the visual quality for the AI and LD configurations between the decoded image of the EVC Baseline profile and the proposed results. The visual quality assessment was performed at a middle QP value as QP = 32. Figure 5 illustrates the comparison results for PartyScene with the AI configuration. The filtered image by post-filtering reveals a superior visual quality compared to the video before post-filtering, aligning with the 0.49 dB improvement observed in the objective evaluation. Notably, the figure of the proposed method in Figure 5 shows a further reduction in artifacts, especially in the face of the child and around the area of the boxes. Similar results are evident in the LD configuration. Figure 6 shows the comparison results for BQTerrace with the LD configuration. In this figure, the filtered image by the proposed method reveals more textural detail than the reference, especially notable in the parasol, where texture lines are clearly observed in the proposed filtered image.

### 4.3. Discussion

The experimental results of the proposed method showed both objective and subjective improvements in performance. In the objective results, the PSNR showed a significant enhancement in both the AI and LD configurations. This PSNR improvement corresponds to a reduced bitrate at the same image quality. Considering the historical fact of video coding standards improving performance by about 50% every decade, achieving a 10% enhancement with just one tool is quite impressive objectively. The subjective experimental results also reveal a remarkable improvement in image quality. The proposed method effectively addresses blocking artifacts, a specific target of enhancement, noticeably eliminating them. Another noteworthy aspect in the subjective experiments is the preservation of details in the original video that typically disappear due to coding artifacts. The proposed method successfully restores these lost details, bringing the visual quality closer to the original video. The significant improvement in visual quality is attributed to the CNN-based post-filter guided by the partitioning map, which identifies areas affected by blocking artifacts and guides the CNN model to enhance these areas in line with the original video.

Nevertheless, it should be noted that implementing the proposed CNN-based method involves considerable complexity, particularly when applied directly to devices within the IoT. The decision to employ a post-filter for enhancing image quality, accounting for diverse computing performances, aligns with the practical challenge of deploying CNN-based filters across the spectrum of IoT devices. In this context, it is believed to be more pragmatic to adaptively apply these filters as add-ons using post-filtering instead of in-loop filtering when external resources are available, as illustrated in the proposed configuration.

### 4.4. Future Work

This paper introduced a CNN-based post-filtering method designed for the EVC Baseline profile to address the requirements of IoT devices. While the proposed method is specifically applied to the EVC Baseline profile, its foundational architecture, shared with other video coding standards, suggests its potential applicability in the EVC Main profile or diverse video codecs. The exploration of extending the method outlined in this paper to other video codecs is considered an interesting topic for future research.

Additionally, it is noted that CNN remains an important role in enhancing the coding performance in this paper. With NNVC in the JVET successfully employing CNN-based deep-learning technology, a similar investigation into CNN-based filtering technology for the EVC Baseline profile has been conducted in the proposed method. Given the novelty of the MPEG-5 EVC Baseline profile and the limited research on leveraging CNN as a post-filter for this codec, exploring this aspect in conjunction with the latest machine-learning techniques is also considered an interesting topic for future research.

## 5. Conclusions

In this paper, a post-filter utilizing a CNN with block partition information for the EVC Baseline profile was proposed. As the demand for efficient video coding technology intensifies, driven by the surge in video data from IoT devices, the EVC Baseline can be considered as a promising solution designed to address the specific requirements of processing IoT video data with low complexity. Nevertheless, enhancements are required to address coding artifacts within the EVC Baseline profile. To tackle this issue, a post-filter utilizing a CNN based on block partition information was introduced in this paper. Through experimental results, both objective and subjective assessments showed significant improvements in both the AI and LD configurations when compared to the pre-post-filter video. The advancements achieved by the proposed method notably enhanced the visual quality, especially in blocking artifacts at the boundaries. Thus, this proposed method is expected to benefit networks of high-performance, low-complexity sensor nodes in IoT ecosystems using the EVC Baseline profile.

## Figures and Tables

**Table 1:** Details of the training environment.

Training dataset	BVI-DVC
Videos	800 videos with 10 frames
Framework	Pytorch 1.13.0
Epoch	50
Optimizer	Adam optimizer with a learning rate of $10^{-4}$
Models	Five models at QP22, 27, 32, 37, and 42 for AI Five models at QP22, 27, 32, 37, and 42 for LD
Anchor encoder	XEVE with Baseline profile setting
Anchor decoder	XEVD with Baseline profile setting
Hardware	AMD EPYC 7513 32-Core CPUs, 384 GB RAM (AMD, Santa Clara, CA, USA), and an NVIDIA A6000 GPU (NVIDIA, Santa Clara, CA, USA).

**Table 2:** Details of the testing environment.

Test dataset	Class A1(4K): Tango2, FoodMarket4, Campfire Class A2(4K): CatRobot, DaylightRoad2, ParkRunning3 Class B(2K): MarketPlace, RitualDance, Cactus, BasketballDrive, BQTerrace Class C(WVGA): BasketballDrill, BQMall, PartyScene, RaceHorses Class D(WQVGA): BasketballPass, BQSquare, BlowingBubbles, RaceHorses
Frames	Full frames
Framework	Pytorch
Models	Five models at QP22, 27, 32, 37, and 42 for AI Five models at QP22, 27, 32, 37, and 42 for LD
Anchor encoder	XEVE with Baseline profile setting
Anchor decoder	XEVD with Baseline profile setting
Hardware	AMD EPYC 7513 32-Core CPUs, 384 GB RAM, and an NVIDIA A6000 GPU.

**Table 3:** Objective testing result of AI configuration.

Class and Sequence		Bitrate (kpbs)	Reference (dB)			Proposed Method (dB)			BD-PSNR (ΔdB)			BD-BR (Δ%)		
			Y-PSNR	U-PSNR	V-PSNR	Y-PSNR	U-PSNR	V-PSNR	ΔY-PSNR	ΔU-PSNR	ΔV-PSNR	ΔY-BDBR	ΔU-BDBR	ΔU-BDBR
A1	Tango2	62,688	38.91	46.67	44.71	39.21	47.74	45.81	0.30	1.07	1.10	−11.42	−41.06	−38.15
	FoodMarket4	121,128	39.01	43.32	44.52	39.53	44.18	45.60	0.52	0.86	1.08	−12.49	−24.31	−29.93
	Campfire	76,616	37.58	39.26	40.26	37.83	40.51	41.07	0.25	1.25	0.81	−6.53	−33.13	−33.41
A2	CatRobot	122,884	37.73	40.35	40.85	38.27	41.08	41.89	0.53	0.73	1.04	−14.76	−36.53	−36.67
	DaylightRoad2	145,191	36.37	43.31	41.39	36.71	44.06	41.74	0.34	0.75	0.35	−11.83	−40.85	−21.43
	ParkRunning3	227,250	38.12	35.40	36.45	38.61	35.61	36.66	0.49	0.21	0.21	−8.34	−5.66	−7.67
B	MarketPlace	42,551	37.16	41.66	42.46	37.54	42.43	43.15	0.38	0.78	0.69	−9.35	−29.12	−28.89
	RitualDance	28,415	39.31	43.95	44.30	40.10	45.05	45.76	0.79	1.10	1.46	−15.27	−33.28	−39.10
	Cactus	47,502	35.36	38.73	40.63	35.84	39.11	41.41	0.48	0.38	0.78	−12.07	−17.94	−28.80
	BasketballDrive	31,843	36.65	42.18	42.70	37.09	42.29	43.31	0.43	0.11	0.61	−11.05	−5.96	−22.38
	BQTerrace	80,937	34.93	40.21	42.31	35.40	40.28	42.45	0.48	0.07	0.13	−7.96	−5.23	−9.76
C	BasketballDrill	11,741	35.27	39.88	39.93	36.25	40.69	41.63	0.98	0.81	1.70	−18.24	−25.33	−40.74
	BQMall	12,610	35.60	40.53	41.45	36.38	41.36	42.60	0.78	0.83	1.15	−14.26	−26.02	−32.56
	PartyScene	22,222	32.96	38.21	38.81	33.45	38.75	39.45	0.49	0.54	0.64	−8.19	−14.89	−16.41
	RaceHorses	7724	35.33	38.58	39.98	35.90	39.61	41.30	0.57	1.03	1.32	−11.12	−27.99	−38.47
D	BasketballPass	2895	35.85	40.78	40.28	36.67	41.92	41.68	0.81	1.14	1.39	−13.41	−28.42	−31.52
	BQSquare	7108	32.98	39.71	40.52	33.75	40.15	41.38	0.77	0.44	0.86	−10.45	−12.45	−23.37
	BlowingBubbles	5886	32.85	37.96	38.37	33.40	38.53	39.16	0.55	0.57	0.79	−9.57	−16.97	−21.52
	RaceHorses	2352	34.74	37.99	39.01	35.63	39.64	40.93	0.89	1.65	1.93	−14.38	−40.42	−46.22
Average									0.57	0.75	0.95	−11.62	−24.50	−28.79

**Table 4:** Objective testing result of LD configuration.

Class and Sequence		Bitrate (kpbs)	Reference (dB)			Proposed Method (dB)			BD-PSNR (ΔdB)			BD-BR (Δ%)		
			Y-PSNR	U-PSNR	V-PSNR	Y-PSNR	U-PSNR	V-PSNR	ΔY-PSNR	ΔU-PSNR	ΔV-PSNR	ΔY-BDBR	ΔU-BDBR	ΔU-BDBR
A1	Tango2	36.94	45.84	43.38	37.17	46.69	44.26	0.23	0.23	0.85	0.88	−8.57	−59.49	−49.19
	FoodMarket4	35.95	41.03	41.92	36.19	42.28	43.40	0.25	0.25	1.26	1.48	−7.01	−63.93	−68.60
	Campfire	35.49	37.26	39.01	35.74	38.11	39.68	0.25	0.25	0.85	0.67	−7.28	−27.81	−34.49
A2	CatRobot	35.28	39.50	39.56	35.61	40.18	40.48	0.33	0.33	0.69	0.93	−10.55	−56.66	−49.27
	DaylightRoad2	33.97	41.81	39.97	34.16	42.75	40.67	0.19	0.19	0.94	0.70	−8.88	−71.13	−58.59
	ParkRunning3	34.05	33.09	34.51	34.30	33.33	34.82	0.26	0.26	0.24	0.31	−5.86	−12.42	−17.60
B	MarketPlace	33.98	40.00	40.89	34.18	40.91	41.63	0.20	0.20	0.91	0.74	−6.59	−63.16	−56.89
	RitualDance	35.71	42.31	42.43	36.14	43.36	43.72	0.43	0.43	1.05	1.29	−9.36	−51.85	−54.47
	Cactus	32.71	37.87	39.64	33.05	38.38	40.37	0.34	0.34	0.50	0.73	−11.87	−48.07	−44.86
	BasketballDrive	33.77	40.74	40.77	34.13	41.39	41.79	0.36	0.36	0.65	1.02	−11.47	−45.44	−48.28
	BQTerrace	31.19	37.83	39.75	31.52	38.72	40.86	0.32	0.32	0.89	1.11	−13.79	−65.73	−70.76
C	BasketballDrill	31.83	37.89	37.72	32.43	39.11	39.20	0.60	0.60	1.22	1.48	−14.77	−48.76	−49.42
	BQMall	31.73	38.74	39.58	32.23	39.87	40.91	0.50	0.50	1.13	1.32	−12.67	−57.01	−58.53
	PartyScene	28.39	36.39	37.13	28.74	36.97	37.67	0.35	0.35	0.58	0.55	−10.83	−29.20	−26.30
	RaceHorses	31.52	36.94	38.55	31.91	37.66	39.53	0.39	0.39	0.72	0.98	−10.72	−41.02	−54.39
D	BasketballPass	31.88	39.26	38.18	32.44	39.91	39.10	0.57	0.57	0.65	0.92	−12.35	−27.59	−32.37
	BQSquare	27.79	38.10	38.60	28.37	38.94	39.74	0.58	0.58	0.84	1.14	−18.51	−60.08	−68.07
	BlowingBubbles	28.37	35.93	36.49	28.68	36.54	37.01	0.31	0.31	0.60	0.52	−9.49	−31.72	−26.01
	RaceHorses	30.77	36.34	37.35	31.31	37.36	38.65	0.54	0.54	1.03	1.30	−12.68	−44.79	−52.78
Average									0.37	0.82	0.95	−10.70	−47.68	−48.47

## Data Availability

Data are contained within the article.
